# Laparoscopic Retrieval of Foreign Body in the Kidney: An Unusual Case Report and Literature Review

**DOI:** 10.1155/criu/8836853

**Published:** 2025-12-19

**Authors:** Abdalatiff K. Bedaiwi, Moath M. Qarmush, Ebtesam Almajed, Khalaf Abdullah Alshamrani, Malek M. Almugharriq, Mohammed Hani Almomen, Khaled K. Bedaiwi

**Affiliations:** ^1^ Department of Urology, Prince Sultan Military Medical City, Riyadh, Saudi Arabia, psmmc.med.sa

**Keywords:** case report, laparoscopy, renal foreign body

## Abstract

**Background:**

Renal foreign bodies are rare clinical entities that may result from iatrogenic injury, ingestion, or trauma. They often present with nonspecific symptoms, making diagnosis challenging.

**Case Presentation:**

We report the case of a 42‐year‐old woman with right flank pain of 2 months and a surgical history of Burch colposuspension and laparoscopic cholecystectomy. Computed tomography revealed a 6‐cm linear hyperdense object, representing a needle‐like foreign body that penetrated the posterior cortex of the right kidney. She underwent successful transperitoneal laparoscopic retrieval, with an uneventful postoperative course and complete recovery.

**Conclusion:**

This case underscores the importance of maintaining a high index of suspicion and supports minimally invasive retrieval as a safe and effective approach, yielding favorable clinical outcomes.

## 1. Introduction

Foreign bodies (FBs) within the urinary system are uncommon, and their presence in the kidney is especially rare [1]. Iatrogenic intrarenal FBs have been reported in the literature, with cases of retained fragments of surgical instruments, guidewires, stents, and biopsy needles. Other less frequent etiologies include traumatic implantation and, rarely, migration of an ingested object into the kidney [2].

Clinically, retained renal FBs may remain asymptomatic for extended periods, with patients often presenting with nonspecific symptoms such as flank pain, hematuria, fever, or recurrent urinary tract infections [1, 3]. These subtle presentations underscore the importance of maintaining a high index of suspicion, especially in patients with a history of surgical interventions.

The clinical importance of retained FBs lies in their potential to trigger significant complications if left unaddressed, including acting as a nidus for infection or stone formation due to encrustation with urinary minerals [4]. Complications may range from fibrous encapsulation to severe inflammation, potentially resulting in abscesses, fistulae, or life‐threatening sepsis [5, 6]. Early recognition and removal are crucial to preventing complications [7].

Herein, we report a 42‐year‐old woman with a needle‐like FB penetrating her right kidney, with a thorough review of the literature. The following case report has been prepared following the Case Reports (CARE) reporting guidelines.

## 2. Case Report

A 42‐year‐old female with a known history of Type 2 diabetes mellitus, hypertension, and psoriasis presented to the emergency department on July 18^th^, 2025, with a 2‐month history of intermittent right flank pain. Her past surgical history was significant for a Burch colposuspension performed in July 2024 and a laparoscopic cholecystectomy 10 years ago. Upon physical examination, her abdomen is soft and lax with mild tenderness over the right flank.

An ultrasound of the right kidney performed in June 2025 revealed no abnormal findings, including the absence of renal stones, hydronephrosis, or FBs. The initial unremarkable sonographic evaluation delayed further investigation until a noncontrast computed tomography (CT) scan was done.

**Figure 1:**
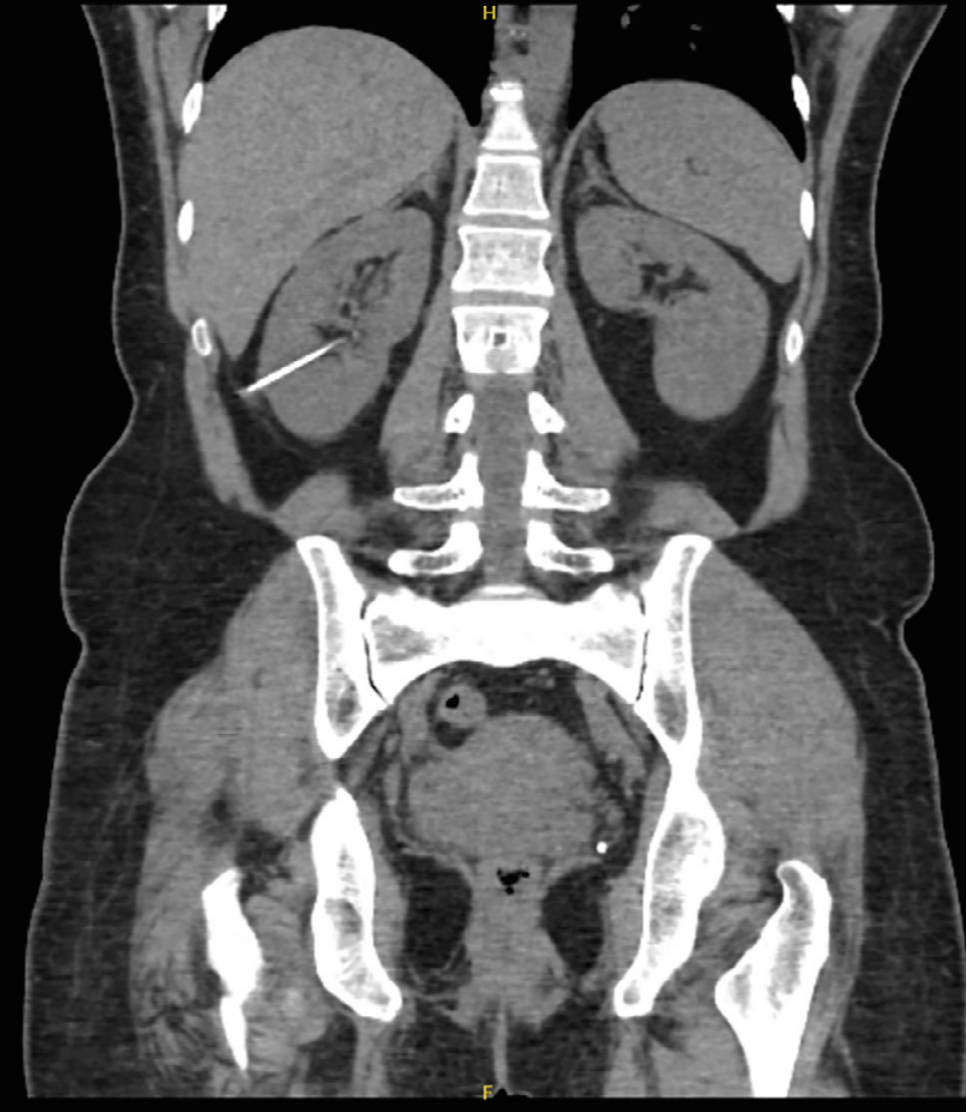
Coronal CT scan with no contrast showing a linear hyperdense foreign body penetrating the posterior cortex of the right kidney and reaching into the lower calyx.

A noncontrast CT scan of the abdomen and pelvis performed using the kidneys, ureters, and bladder (KUB) protocol demonstrated no nephrolithiasis or hydronephrosis. However, a linear hyperdense structure, consistent with a needle‐like FB measuring approximately 6 cm in length, was seen penetrating the posterior cortex of the right renal lower pole and extending into the lower calyx (Figure 1). No perinephric fat stranding, hematoma, or collection was observed. Additionally, a stable, exophytic 2.2 cm right upper pole renal cyst was incidentally identified.

**Figure 2:**
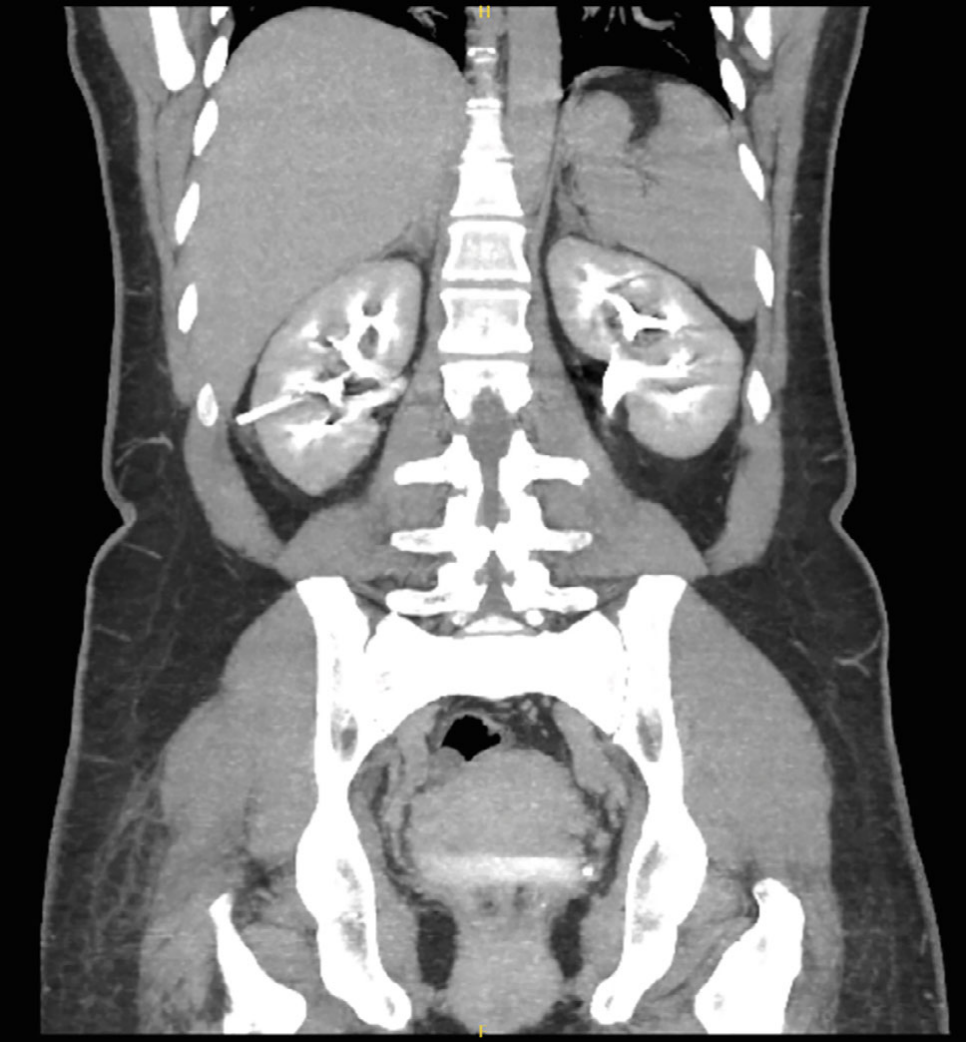
CT urography showing no contrast extravasation, urinary leak, or vascular injury.

To assess potential injury to the renal vasculature or collecting system, a triphasic CT urography was done. It showed no contrast extravasation, urinary leak, or vascular injury (Figure 2).

**Figure 3:**
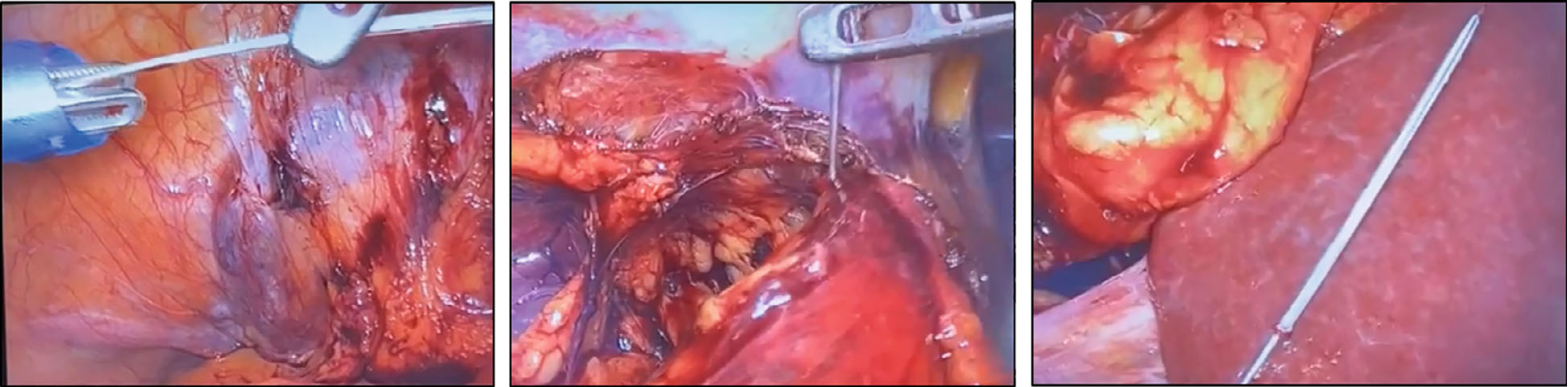
Laparoscopic images of the retained needle embedded in the right kidney.

**Figure 4:**
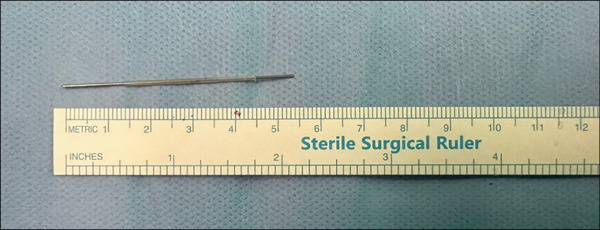
The retrieved metallic foreign body measured approximately 5.5 cm in length.

Considering these findings, the patient underwent transperitoneal laparoscopic exploration. Under general anesthesia in the left lateral decubitus position with standard skin preparation and draping, pneumoperitoneum was established using the open Hasson technique. An 11‐mm camera port was inserted under direct vision, followed by the insertion of two additional 5‐mm working ports. Intraoperatively, dense adhesions were observed between the bowel loops and the lower pole of the right kidney, which were carefully dissected and released to expose the lateral aspect of the renal surface. The metallic needle was identified embedded within the posterior cortex of the lower pole of the right kidney. Using laparoscopic graspers, the FB was successfully retrieved without complications (Figures 3 and 4). Despite the thorough review of the patient′s surgical history and operative records, no definitive source of the FB could be established. The postoperative course was uneventful, and the patient was discharged in good condition after a 2‐day hospital stay.

**Figure 5:**
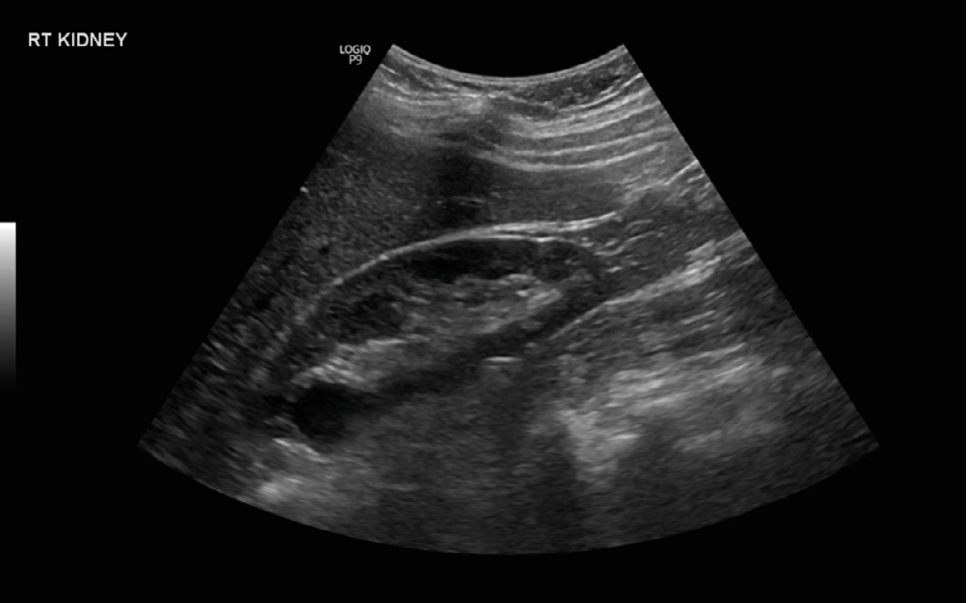
Right kidney ultrasound demonstrating normal size and echogenicity with preserved corticomedullary differentiation.

Postoperative follow‐up imaging was performed 2 months after surgery. A renal ultrasound demonstrated both kidneys to be of normal size and echogenicity with preserved corticomedullary differentiation (Figure 5). The previously noted right renal FB was no longer visible, and there was no evidence of hydronephrosis, focal lesion, or perinephric collection. The urinary bladder was unremarkable, with no significant postvoid residual volume. These findings confirmed complete resolution and absence of postoperative complications.

## 3. Discussion

Retained FBs within the kidney are infrequently reported in the literature and represent a rare clinical entity, with variable routes of entry and potential complications. A thorough review of the literature identified fewer than 30 published cases of renal FBs over the past several decades, with 10 reported within the last decade [1, 2, 4–11]. Intrarenal FBs are mostly documented as isolated case reports rather than in large series, highlighting their rarity. These cases vary widely in etiology, presentation, imaging findings, and management strategies. To provide a contemporary perspective, we focused on the most recent decade. Table 1 highlights the clinical characteristics, diagnostic modalities, interventions, and outcomes of renal FB cases published over the last decade.

**Table 1:** Overview of case reports reporting renal foreign bodies in the last decade.

**Study**	**Age**	**Gender**	**Side**	**Object type**	**Etiology**	**Symptoms**	**Indwelling period**	**Management**	**Outcome**
Almuallem et al., 2015 [8]	4	Male	Right	Bobby pin (6 cm)	Ingestion	RUQ pain, flank pain, fever, chills	3 mo	Laparotomy with duodenal repair	Uneventful recovery
Upadhyay et al., 2015 [9]	20	Male	Right	3 metallic objects	Ingestion	Flank pain, gross hematuria	1 year	Retrograde percutaneous nephrostomy	Uneventful recovery
Tüdös et al., 2016 [11]	43	Male	Right	Wooden toothpick	Ingestion	Flank pain, gross hematuria	5 mo	URS	Uneventful recovery
Guo et al., 2017 [10]	53	Male	Right	Iron wire (10 cm)	Suicidal ingestion	Asymptomatic	6 mo	Conservative, as the patient refused	Uneventful 30‐month follow‐up
Mamoulakis et al., 2018 [4]	65	Male	Right	Nephrostomy locking suture	Iatrogenic	Asymptomatic	19 mo	RIRS and laser	Uneventful recovery
Jamil et al., 2020 [2]	18	Female	Right	Rubber drain tube	Iatrogenic	Flank pain, nausea	2 mo	PCNL	Uneventful recovery
Farshi & Jafarlou, 2020 [5]	34	Male	Left	Sewing needle (5 cm)	Unknown	Left flank pain	2 mo	Laparoscopic transperitoneal retrieval	Uneventful recovery
Zhang et al., 2023 [6]	22	Male	Right	Wooden toothpick (6 cm)	Ingestion	Abdominal pain	10 years	Extracorporeal ultrasound‐assisted endoscopic duodenum	Uneventful recovery
Sommer & Thalmann, 2023 [1]	77	Male	Right	Wooden toothpick	Ingestion	Recurrent UTI, fever, chills, flank pain	Unknown	URS	Uneventful recovery
Gopi et al., 2025 [7]	9mo	Male	Left	Broken hypodermic needle (3 cm)	Iatrogenic	Asymptomatic (incidental finding)	9 mo	Laparoscopic retrieval	Uneventful recovery
Bedaiwi et al., 2025	42	Female	Right	Needle‐like foreign body (6 cm)	Unknown with the possibility of being iatrogenic	Intermittent right flank pain	12 mo	Laparoscopic transperitoneal retrieval	Uneventful recovery

Abbreviations: mo, months; PCNL, percutaneous nephrolithotomy; RIRS, retrograde intrarenal surgery; RUQ, right upper quadrant; URS, ureteroscopy.

A retrospective analysis of retained items revealed that sponges accounted for approximately 69% of cases, while sharps and instruments accounted for the remaining 31% [3]. Needles pose unique challenges due to their size and potential to migrate. Our case contributes to the limited literature, highlighting that even in the modern era of preventive measures, such events can still occur.

Once a FB is suspected, a CT scan is the diagnostic modality of choice, offering excellent anatomical detail for precise localization within the renal parenchyma or collecting system, as well as for identifying associated reactive changes. Furthermore, CT urography is particularly valuable in delineating the relationship between the foreign object, the renal collecting system, and adjacent vasculature. Nonmetallic FBs, such as cloth or wood, may produce subtle findings that often mimic mass‐like lesions and can be misdiagnosed as tumors. Mamoulakis et al. reported a unique case in which a calcified nephrostomy tube (NT) locking suture remnant simulated a renal pelvic tumor on MRI nearly 2 years after initial NT placement [4]. The case underscores the diagnostic challenges posed by intrarenal FBs, especially when patients lack overt symptoms. Similarly, Dogra et al. described a suture granuloma that radiologically resembled renal cell carcinoma and resulted in an unnecessary laparoscopic nephrectomy [12].

Among intrarenal FB routes, gastrointestinal migration is particularly noteworthy. Baird and Spence reported ingested needles perforating the duodenum into the kidney, causing delayed complications [13]. A recent pediatric case of a bobby pin penetrating the duodenum and renal parenchyma presented similarly, with recurrent urinary tract infections and flank pain [8].

Once a retained renal FB is identified, removal is typically indicated, given the risks of leaving it in place [7]. The retrieval method should be guided by the object′s characteristics and the patient′s condition. Historically, renal FBs often required radical nephrectomy as documented by Osmond′s mid‐20th‐century review [14]. However, advances in endourology have significantly shifted the management paradigm. Techniques such as percutaneous nephrolithotomy and laparoscopy now enable effective, organ‐preserving removal with reduced morbidity. Singh et al. reported the successful laparoscopic retrieval of a malleable copper wire embedded in renal parenchyma [15]. Farshi and Jafarlou further underscore the efficacy of laparoscopy for extracting a deeply embedded sewing needle [5]. Similarly, Gopi and Savio achieved laparoscopic removal of a 3‐cm needle from an infant′s kidney, avoiding open surgery [7]. In our patient, the decision was made to proceed with a laparoscopic approach, which contributed to her smooth recovery. These cases illustrate the shift toward minimally invasive approaches, supported by their proven safety, efficacy, and favorable outcomes.

## 4. Conclusion

Renal FBs remain a rare clinical finding, often presenting with nonspecific symptoms and posing diagnostic challenges. This case highlights the importance of maintaining a high index of suspicion, especially in patients with a relevant surgical history. Our case, alongside the reviewed literature, underscores the growing preference for a minimally invasive approach that offers effective FB retrieval with excellent patient outcomes.

## Consent

Written informed consent was obtained from the patient for publication of this case report and any accompanying images.

## Conflicts of Interest

The authors declare no conflicts of interest.

## Funding

The authors received no specific funding for this work.

## Data Availability

The data that support the findings of this study are available from the corresponding author upon reasonable request.
